# Limb‐bud and heart development (LBH) contributes to glioma progression *in vitro* and *in vivo*

**DOI:** 10.1002/2211-5463.13325

**Published:** 2021-11-26

**Authors:** Luotong Liu, Qinglian Luo, Qian Xu, Yu Xiong, Huajiang Deng

**Affiliations:** ^1^ Department of Neurosurgery Affiliated Hospital of Southwest Medical University Luzhou China

**Keywords:** glioma, progression, LBH, migration, invasion

## Abstract

Glioma is the predominant brain malignancy and is correlated with high mortality and severe morbidity. The transcription factor limb‐bud and heart (LBH) has been reported to be involved in the development of several cancers, although its role in glioma development remains elusive. Here, we examined the effect of LBH on glioma progression. The expression of LBH was increased in glioma samples from The Cancer Genome Atlas database, and upregulation of LBH was observed to be correlated with the poor survival of glioma patients. We also report that expression of LBH was elevated in clinical glioma tissues compared to adjacent normal tissues, and was also enhanced in glioma cell lines. LBH promotes proliferation and inhibits cell cycle arrest and apoptosis in glioma cells. In addition, LBH increased the migration and invasion of glioma cells *in vitro*. Moreover, tumorigenicity analysis revealed that LBH could promote the tumor growth of glioma cells *in vivo*. In conclusion, our findings suggest that LBH contributes to glioma progression *in vitro* and *in vivo*. Our findings provide new insights into the mechanism by which LBH promotes the development of glioma, improving our understanding of the correlation between LBH with cancer. LBH may have potential as a target for glioma therapy.

## Graphical abstract

Limb‐bud and heart (LBH) contributes to glioma progression *in vitro* and *in vivo*. Our findings provide new insights into the mechanism by which LBH promotes the development of glioma, improving our understanding of the correlation between LBH with cancer. LBH may have potential as a target for glioma therapy.

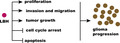

**Abbreviations**
BrdU: bromodeoxyuridine
LBH: limb‐bud and heart
MTT: 3‐[4,5‐dimethylthiazol‐2‐yl]‐2,5‐diphenyl tetrazolium bromide
PBS: phosphate‐buffered saline
qRT‐PCR: quantitative reverse transcriptase‐polymerase chain reaction
shRNA: short hairpin RNA

Human glioma is the most prevalent type of intracranial malignancy with high mortality and a poor prognosis [1]. Glioma has been numerically graded (I–IV) for the pathologic characteristics of the tumor by the World Health Organization [2]. Moreover, glioma is a typical human brain malignant with poor survival and considerable morbidity [3, 4]. The current treatment of glioma relies on the location, grade, type and size of the tumor, as well as the overall health and age of the cases. It principally consists of curative resection guided by different therapy, such as gene therapy, chemotherapy and radiotherapy [5, 6]. Nevertheless, the recurrence or death of glioma remains unsatisfactory as a result of metastasis and drug resistance [7, 8], and the complicated development of glioma is still to be established, comprising aberrant activation of tumor suppressors and oncogenes [9, 10]. Accordingly, it is essential to improve the understanding of the molecular mechanism and develop significant targets for glioma diagnosis and treatment.

Limb‐bud and heart development (LBH) is a profoundly tissue‐specific and conserved transcription co‐activator in mammals in which LBH modulates various essential genes during embryonic development [11, 12]. LBH can express in the organs of adults, such as the peripheral nervous system, brain, kidney, gut and spleen, except embryonic tissues [13]. The abnormal expression of LBH throughout cardiac development is able to cause fundamental cardiac disorders, including growth defects and partial trisomy 2p syndrome [12, 13, 14, 15, 16, 17]. Growing evidence shows that tumorigenesis and embryonic development possess related molecular mechanisms [18]. It has been reported that LBH, as a novel cancer‐related factor, plays crucial roles in the modulation of lung adenocarcinoma [19], prostate cancer [20], hepatocellular carcinoma [21] and nasopharyngeal carcinoma [22]. LBH serves as a targeted factor of Wnt signaling to increase Wnt‐related cancer progression and repress mammary epithelial differentiation [23]. Moreover, LBH overexpression enhances angiogenesis by regulating the vascular endothelial growth factor A‐related extracellular signal‐regulated kinase pathway in human glioma under hypoxia conditions [24]. However, the expression status and biological function of LBH in glioma, especially the role of LBH in the modulation of glioma malignant progression, remain unclear.

In the present study, we were interested in the role LBH in the pathogenesis of glioma. We identified a novel function of LBH with respect to promoting glioma progression *in vitro* and *in vivo*.

## Materials and methods

### Glioma clinical samples

The 10 glioma clinical samples used in the present study were obtained from the Affiliated Hospital of Southwest Medical University. All of the patients were diagnosed with a glioblastoma by histopathological analysis. All cases were independently diagnosed and reviewed by two clinicians. Before surgery, no systemic or local therapy was carried out in the subjects. The glioma tissues and corresponding para‐neoplastic tissues obtained from the patients were immediately frozen in liquid nitrogen, followed by storage at −80 °C before further analysis. The glioma samples were also obtained from The Cancer Genome Atlas (TCGA) database (https://www.cancer.gov/about‐nci/organization/ccg/research/structural‐genomics/tcga). Written informed consent was obtained from each subject in the present study. All experiments conform to the Declaration of Helsinki, and the study methodologies were approved by the appropriate local ethics committee and the Ethics Committee of Affiliated Hospital of Southwest Medical University.

### Cell culture and treatment

The normal human bronchial epithelial HBE cell line and the human glioblastoma SHG44, U87 and U251 cell lines were obtained from the American Type Tissue Culture Collection (Manassas, VA, USA). The cells were cultured in Dulbecco’s modifed Eagle’s medium (Gibco, Amarillo, TX, USA) containing 100 units·mL^−1^ penicillin (Gibco), 0.1 mg·mL^−1^ streptomycin (Gibco) and 10% FBS (Gibco) at 37 °C with 5% CO_2_. The lentivirus vector containing LBH short hairpin RNA (shRNA) and pcDNA3.1‐LBH overexpression vector and the corresponding controls were obtained from GenePharma (Suzhou, China). The transfection in the cells was performed using Liposome 3000 (Invitrogen, Waltham, MA, USA) in accordance with the manufacturer’s instructions.

### Colony formation assays

About 1 × 10^3^ cells were layered in six wells and incubated in Dulbecco’s modifed Eagle’s medium at 37 °C. After 2 weeks, cells were cleaned with phosphate‐buffered saline (PBS) in methanol for approximately 30 min, and dyed with crystal violet dye at a dose of 1%, after which the number of colonies was calculated.

### Bromodeoxyuridine (BrdU) incorporation assays

The cell proliferation was assessed via BrdU incorporation assays. About 2 × 10^3^ cells were put into 96 wells and cultured for 12 h. Then, the cells were used for the transfection or treatment. After 96 h, cell proliferation was measured using a BrdU Cell Proliferation Assay Kit (Sigma, St. Louis, MO, USA).

### 3‐[4,5‐Dimethylthiazol‐2‐yl]‐2,5‐diphenyl tetrazolium bromide (MTT) assays

MTT assays measured the cell viability of U87 and U251 cells. Briefly, approximately 1 × 10^4^ U87 and U251 cells were put into 96 wells and cultured for 12 h. After treatment, the cells were added with a 10 μL of MTT solution (5 mg·mL^−1^; Sigma, St Louis, MO, USA) and cultured for an extra 4 h. Discarded medium and 150 μL·well^−1^ DMSO (Thermo Fisher, Waltham, MA, USA) were used to treat the wells. An ELISA browser was applied to analyze absorbance at 570 nm (EL 800; Bio‐Tek, Winooski, VT, USA).

### Cell cycle analysis

Approximately 1 × 10^5^ cells were plated on six‐well dishes and treated as indicated. Floating and adherent cells were fixed in cold ethanol (4 °C; 70% in PBS) overnight. RNaseA (100 μg·mL^−1^) was added to the cells at 37 °C for 30 min, followed by propidium iodide staining (50 μg·mL^−1^; 30 min) in the dark and flow cytometric analysis using FACSCalibur cytometry (Becton Dickinson, Franklin Lakes, NJ, USA). Approximately 10,000 events were calculated for each sample and the distribution of cell cycle was analyzed using cell quest (Becton Dickinson).

### Analysis of cell apoptosis

Around 2 × 10^5^ cells were plated on six‐well dishes. Cell apoptosis was measured using an Annexin V‐FITC Apoptosis Detection Kit (Cell Signaling Technology, Danvers, MA, USA) in accordance with the manufacture’s instruction. Briefly, approximately 2 × 10^6^ washed cells were collected by binding buffer and then incubated with 1 × FITC‐conjugated Annexin V and propidium iodide solution at room temperature for 20 min in the dark, followed by flow cytometry analysis.

### Transwell assays

To analyze the cell migration, the cells were cultured for 24 h and resuspended in serum‐free culture medium, and then plated into the apical chamber of a Transwell insert at a density of 5 × 10^3^ cells·well^−1^. The culture medium was made up to 150 μL, and 600 μL of complete culture medium was added to the basolateral chamber. After 24 h of culture at 37 °C and 5% CO_2_, the cells were fixed with 4% paraformaldehyde for 10 min and stained with crystal violet dye for 20 min, followed by the analysis using bio imaging navigator (Olympus, Tokyo, Japan). The migrated cells were recorded and calculated.

To analyze the cell invasion, Matrigel was melted overnight at 4 °C and diluted with precold serum‐free culture medium (ratio 8 : 1). The medium (50 μL) was plated into the Transwell polycarbonate membrane with a pore diameter of 8 μm, ensuring that all of the wells were covered with Matrigel at 37 °C for 2 h. The cells were cultured for 24 h and resuspended in serum‐free culture medium and then plated into the apical chamber of the Transwell insert at 1 × 10^5^ cells·well^−1^, and the medium was made up to 150 μL. Then, 600 μL of complete medium with 50% FBS was added with to the basolateral chamber. After 24 h, the cells were fixed using 4% paraformaldehyde for 15 min and stained with crystal violet dye for 10 min. The invaded cells were analyzed and calculated.

### Wound healing assay

The U87 and U251 cells were put in the 24‐well plate at 3 × 10^5^ per well and cultured overnight to reach full confluence as a monolayer. A 20‐μL pipette tip was applied to slowly cut a straight line across the well. Next, the well was washed with PBS three times and changed to serum‐free medium and then allowed to continue to culture. The wound healing percentage was calculated.

### Quantitative reverse transcriptase‐polymerase chain reaction (qRT‐PCR)

The total RNAs were extracted by Trizol (Invitrogen) from the tissues and cells. The first‐strand cDNA was synthesized using a First‐Strand cDNA Synthesis Kit (Thermo Fisher) in accordance with the manufacturer's instruction. The qRT‐PCR was carried out by using a SYBR Real‐time PCR I kit (Takara, Shiga, Japan). The standard control for mRNA was GAPDH. Quantitative determination of the RNA levels was conducted using SYBR GreenPremix Ex TaqTM II Kit (Takara). The experiments were independently repeated at least three times. The primer sequences were:

LBH F: 5′‐GCCCCGACTATCTGAGATCG‐3′

LBH R: 5′‐GCGGTCAAAATCTGACGGGT‐3′

GAPDH F: 5′‐AAGAAGGTGGTGAAGCAGGC‐3′

GAPDH R: 5′‐TCCACCACCCAGTTGCTGTA‐3′

### Western blot analysis

Total proteins were extracted from the cells or mice tissues with RIPA buffer (Cell Signaling Technology). Protein concentrations were measured by using a BCA Protein Quantification Kit (Abbkine, Waltham, MA, USA). The same concentration of protein was divided by SDS/PAGE (12% polyacrylamide gels) and transferred to PVDF membranes (Millipore, Burlington, MA, USA) in a subsequent step. The membranes were hindered with 5% milk and hatched overnight at 4 °C with the primary antibodies for LBH (dilution 1 : 1000; Abcam, Cambridge, MA, USA) and β‐actin (dilution 1 : 1000; Abcam) in which β‐actin served as the control. Then, the corresponding second antibodies (dilution 1 : 1000; Abcam) were used for hatching the membranes for 1 h at room temperature, followed by visualization using an Odyssey CLx Infrared Imaging System (LI‐COR Biosciences, Lincoln, NE, USA).

### Analysis of tumorigenicity in nude mice

The effect of LBH on tumor growth *in vivo* was analyzed in nude mice of Balb/c (male, 4 weeks old; *n* = 6). The U251 cells were treated with shRNA control or LBH shRNA. Approximately 1 × 10^7^ cells were subcutaneously injected in the mice. After 7 days of injection, tumor growth was measured every 7 days. The mice were killed after 35 days of injection and tumors were scaled. Tumor volume was observed by estimating the length and width with calipers and measured with the method of 0.5 × width^2^ × length. The expression levels of Ki‐67 and LBH in the tumor tissues were tested by immunohistochemical staining with the antibodies. The animal care and procedural methods were authorized by the Animal Ethics Committee of Affiliated Hospital of Southwest Medical University.

### Statistical analysis

Data are presented as the mean ± SD. Statistical analysis was conducted using prism, version 7 (GraphPad Software Inc., San Diego, CA, USA). An unpaired Student’s *t*‐test was used to compare two groups and the one‐way analysis of variance followed by Tukey’s post‐hoc test was utilized for comparisons between multiple groups. *P* < 0.05 was considered statistically significant.

## Results

### The expression of LBH is elevated in glioma samples and associated with the poor survival of glioma patients

**Fig. 1:**
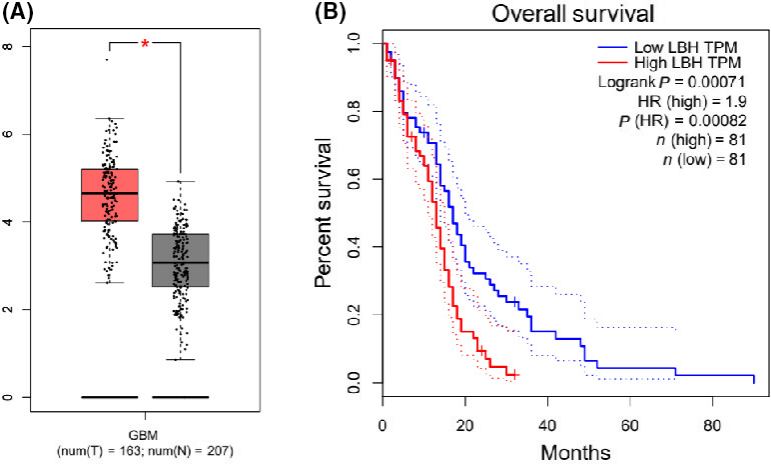
The expression of LBH is elevated in glioma samples and associated with the poor survival of glioma patients. (A) The expression levels of LBH were analyzed in glioma samples from TCGA database. (B) The correlation of LBH with the survival outcomes was analyzed in glioma samples from TCGA database. Student’s *t*‐test: statistically significant differences are indicated (*P < 0.05).

To assess the potential correlation of LBH with glioma progression, we analyzed the LBH expression in glioma samples from TCGA database. Significantly, the levels of LBH were increased in glioma samples relative to normal samples (Fig. 1A). Moreover, the survival analysis showed that the upregulation of LBH is closely correlated with the poor survival of glioma patients (Fig. 1B). Together, these data imply that the abnormal expression of LBH may contribute to the glioma progression.

### The expression of LBH is increased in clinical glioma patients

**Fig. 2:**
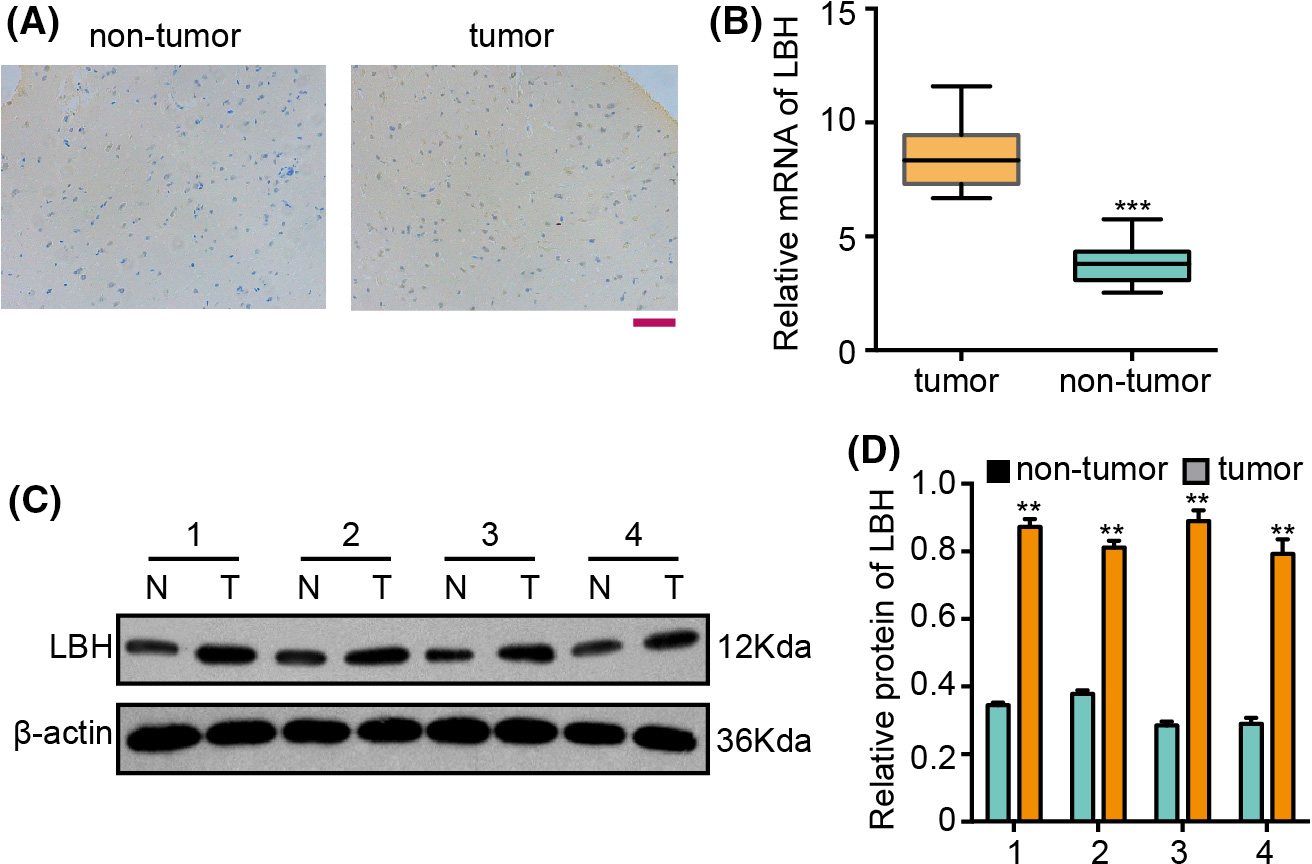
The expression of LBH is increased in clinical glioma patients. (A) The expression levels of LBH were analyzed by immunohistochemical staining in glioma samples. (B) The mRNA expression of LBH was measured by a qPCR in clinical glioma tissues (*n* = 10) and adjacent normal tissues (*n* = 10). (C, D) The protein expression of LBH was assessed by western blot analysis in clinical glioma tissues and adjacent normal tissues. The results of western blot analysis were quantified using imagej. Scale bar = 100 μm. Student’s *t*‐test (*n* = 3). Data are presented as the mean ± SD. Statistically significant differences are indicated (**P < 0.01, ***P < 0.01).

Immunohistochemical staining showed that LBH was positive in the glioma tissues of patients (Fig. 2A). Moreover, mRNA expression was elevated in clinical glioma tissues (*n* = 10) compared to adjacent normal tissues (*n* = 10) (Fig. 2B). Similarly, western blot analysis validated that the protein expression of LBH was upregulated in the clinical glioma tissues relative to adjacent normal tissues (Fig. 2C,D).

### LBH promotes proliferation and inhibits cell cycle arrest and apoptosis in glioma cells

**Fig. 3:**
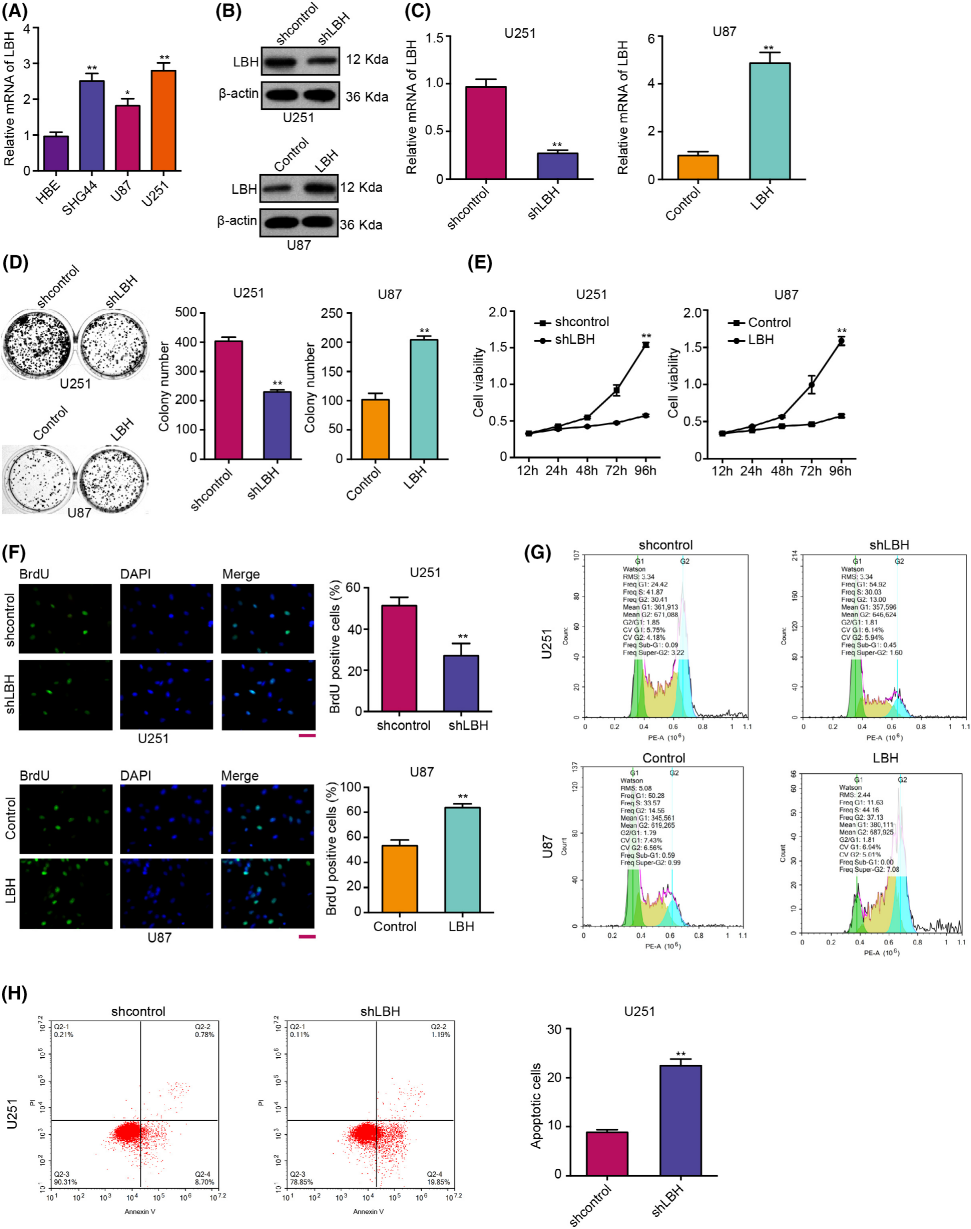
LBH promotes proliferation and inhibits cell cycle arrest and apoptosis in glioma cells. (A) The expression of LBH was measured by a qPCR in SHG44, U87, U251 and HBE cells. (B, H) U251 cells were treated with control shRNA or LBH shRNA, and U87 cells were treated with control vector or LBH overexpression vector. (B) mRNA expression of LBH was assessed by a qPCR. (C) Protein expression of LBH was tested by western blot analysis. (D) Cell proliferation was measured by colony formation assays. (E) Cell viability was determined by MTT assays. (F) Cell proliferation was tested by BrdU incorporation assays. (G) The cell cycle was analyzed by flow cytometry analysis. (H) Cell apoptosis was measured by flow cytometry analysis. Scale bar = 100 μm. Student’s *t*‐test (*n* = 3). Data are presented as the mean ± SD. Statistically significant differences are indicated (**P < 0.01).

We further explored the effect of LBH on the progression of glioma cells. First, we found that LBH was upregulated in glioma cells, including SHG44, U87 and U251 cells, compared to normal HBE cells (Fig. 3A). Then, U251 cells were treated with LBH shRNA and U87 cells were treated with LBH overexpression vector, and the efficiency of LBH shRNA and LBH overexpression vector was validated in the cells (Fig. 3B,C). Significantly, colony formation assays revealed that the colony numbers were decreased by LBH depletion, whereas they were increased by LBH overexpression in the cells (Fig. 3D). Similarly, depletion of LBH reduced, whereas overexpression of LBH enhanced, cell viability in the cells (Fig. 3E). Meanwhile, BrdU incorporation assays demonstrated that proliferation of glioma cells was inhibited by LBH knockdown and promoted by LBH overexpression (Fig. 3F). Moreover, G0/G1 phase cells were enhanced, whereas S phase and G2/M phase cells were reduced, by depletion of LBH in the cells, although the overexpression of LBH showed a reversed effect (Fig. 3G), suggesting that LBH was able to inhibit cell cycle arrest at the G0/G1 phase in glioma cells. In addition, the depletion of LBH could induce apoptosis of U251 cells (Fig. 3H). Together, these data suggest that LBH promotes proliferation and inhibits cell cycle arrest and apoptosis in glioma cells.

### LBH promotes invasion and migration of glioma cells

**Fig. 4:**
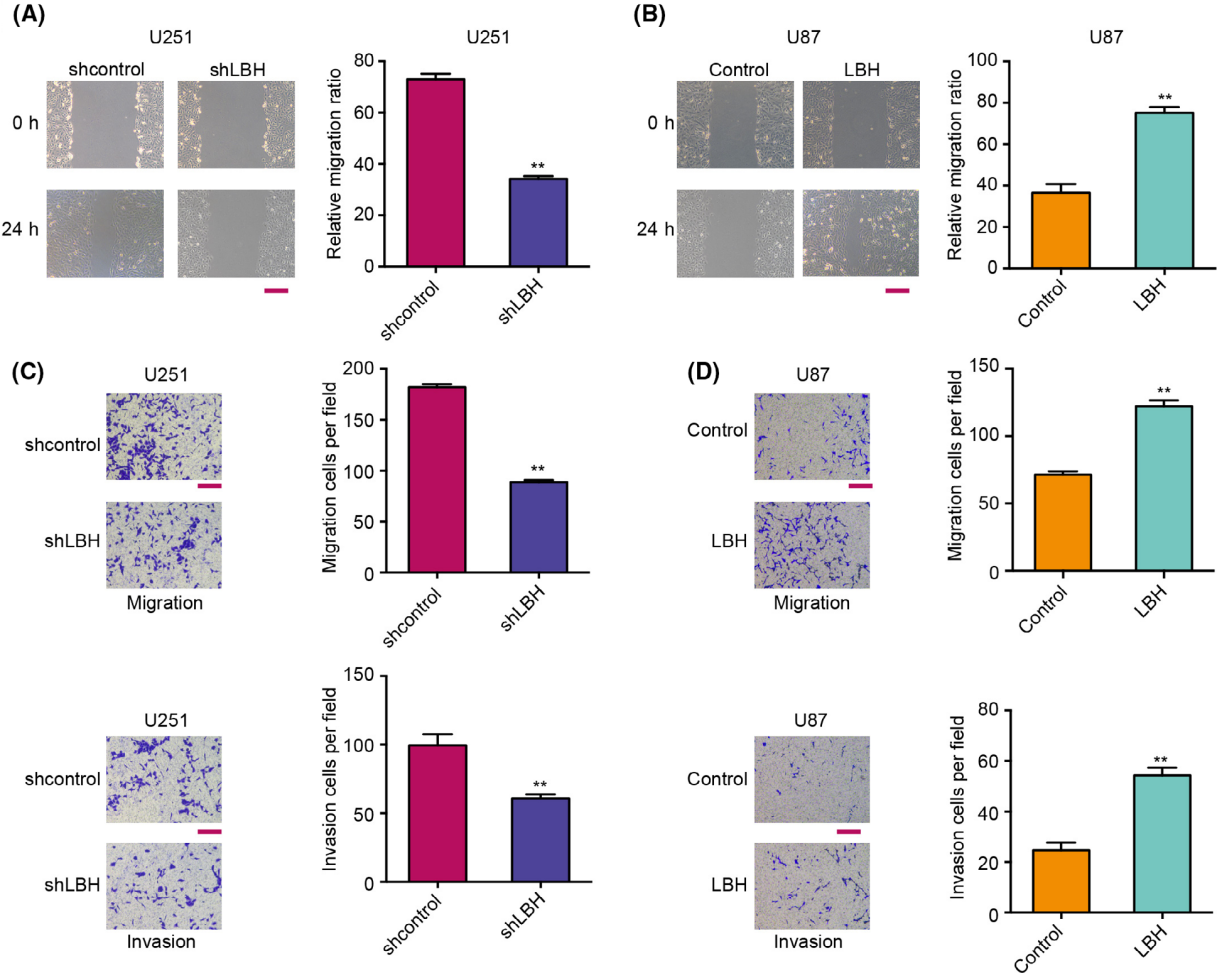
LBH promotes the invasion and migration of glioma cells. (A–D) U251 cells were treated with control shRNA or LBH shRNA, and U87 cells were treated with control vector or LBH overexpression vector. (A, B) Cell migration and invasion were examined by Transwell assays. (C, D) Cell migration was analyzed by wound healing assays. Scale bar = 100 μm. Student’s *t*‐test (*n* = 3). Data are presented as the mean ± SD. Statistically significant differences are indicated (**P < 0.01).

Next, we evaluated the role of LBH in the modulation of migration and invasion in the glioma cells. Transwell assays revealed that migration and invasion of glioma cells were significantly reduced by LBH depletion but enhanced by LBH overexpression (Fig. 4A,B). Similarly, depletion of LBH increased, whereas overexpression of LBH decreased, wound proportion in the cells (Fig. 4C,D), suggesting that LBH promotes the migration and invasion of glioma cells *in vitro*.

### LBH contributes to tumor growth of glioma *in vivo*

**Fig. 5:**
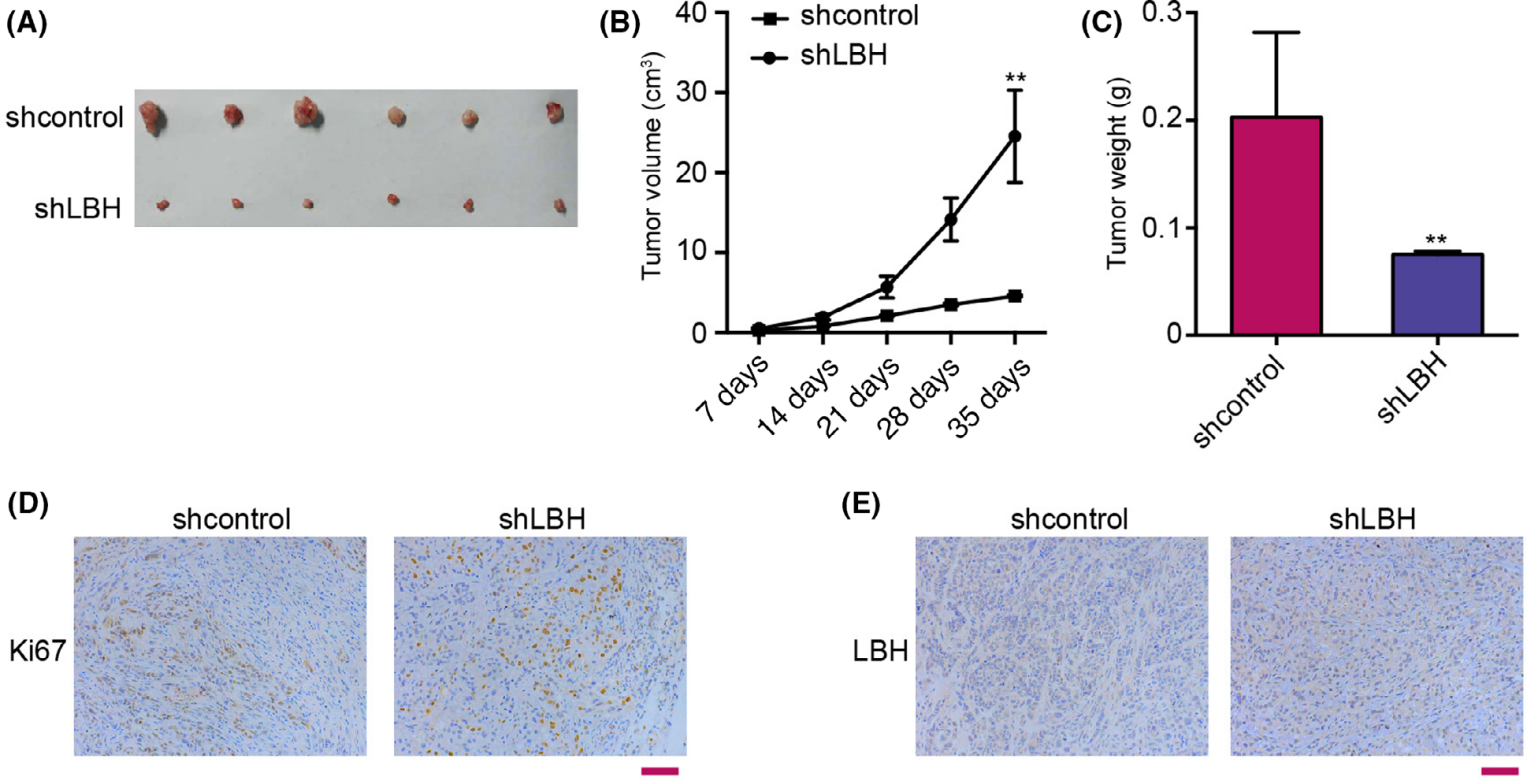
LBH contributes to the tumor growth of glioma *in vivo*. (A–E) The effect of LBH on tumor growth of glioma cells *in vivo* was analyzed by a nude mice tumorigenicity assay. U251 cells were treated with LBH shRNA or shRNA control. U251 cells were subcutaneously injected in mice (*n* = 6). (A) Representative images of dissected tumors from nude mice are presented. (B) Average tumor volume was calculated. (C) Average tumor weight was calculated. Data are presented as the mean ± SD. Statistically significant differences are indicated (**P < 0.01). (D, E) Protein expression levels of Ki67 and LBH were examined by immunohistochemical staining in tumor tissues. Scale bar = 100 μm. Student’s *t*‐test (*n* = 6).

Next, we determined the impact of LBH in the glioma development *in vivo*. For this purpose, tumorigenicity analysis was performed in nude mice injected with U251 cells treated with LBH shRNA. Our data showed that the depletion of LBH remarkably attenuated tumor growth of U251 cells *in vivo*, as demonstrated by tumor size (Fig. 5A), tumor volume (Fig. 5B) and tumor weight (Fig. 5C), as well as the decreased expression of Ki‐67 (Fig. 5D). Furthermore, the expression of LBH was reduced by LBH knockdown in the tumor tissues of mice (Fig. 5E). Together, these results suggest that LBH is able to promote tumor growth of glioma *in vivo*.

## Discussion

Glioma is the predominant type of brain cancer with severe morbidity and high mortality [2]. LBH is a cancer‐associated factor and participates in the development of multiple cancer models. Nevertheless, the role of LBH in the glioma progression remains unclear. In the present study, we found that LBH contributed to the malignant progression of ovarian cancer both *in vitro* and *in vivo*.

It has been confirmed that aberrantly expressed genes participate in the development of glioma. For example, it was reported that MeCP2 is elevated in glioma, and the overexpression of MeCP2 represses invasion, migration and proliferation by regulating the extracellular signal‐regulated kinase pathway in glioma [25]. The expression of IL13Rα2 is correlated with gene expression of the mesenchymal signature and poor prognosis of patients [26]. The expression of HDAC3 is associated with the grade and prognosis of glioma patients [27]. Enhanced pituitary tumor‐transforming gene‐1 (PTTG1) expression correlates with the unsatisfactory prognosis in patients with glioma [28]. PLOD2 is upregulated in glioma samples and increases invasion, migration and proliferation of glioma cells by modulating the phosphoinositide 3‐kinase/Akt pathway [29]. Suppression of PPP3CC by ZEB1 contributes to nuclear factor‐kappa B activation and promotes the growth and invasion of glioma cells [30]. In the present study, we found that the expression of LBH was elevated in glioma tissues, glioma samples from TCGA database and glioma cells. This implies that the abnormally expressed LBH in glioma may be involved in the progression of glioma in clinical context, serving as a potential biomarker of glioma.

Upregulated expression of LBH is correlated with poor prognosis and enhanced invasion and proliferation of gastric cancer cells as a result of upregulating integrin/focal adhesion kinase/Akt signaling [31]. LBH elevation is correlated with the poor prognosis of liver cancer patients [32]. LBH inhibits lung cancer cell invasion and growth and prophesizes survival outcomes [19].

LBH is a new marker for gastric intestinal‐type adenocarcinoma [33]. The enhancement of LBH represses the migration and proliferation in prostate cancer [34]. The Wnt pathway directly targets LBH in aggressive basal subtype breast cancers and epithelial development [35]. In the present study, we revealed that LBH could enhance migration, invasion and proliferation and also attenuate apoptosis in glioma cells. LBH promoted tumor growth of glioma *in vivo*. These data present a novel function of LBH in glioma progression, providing valuable evidence for the fundamental role of LBH in the development of cancer.

In conclusion, we have revealed that LBH contributed to glioma progression both *in vitro* and *in vivo*. Our findings provide new insights into the mechanism by which LBH promotes the development of glioma, improving our understanding of the correlation of LBH with cancer. LBH may serve as a potential target for glioma therapy.

## Conflict of interests

The authors declare that they have no conflicts of interest.

## Author contributions

Huajiang Deng and Luotong Liu conceived and designed the project. Luotong Liu, Qinglian Luo, Qian Xu and Yu Xiong acquired the data. Luotong Liu, Qinglian Luo, Qian Xu and Yu Xiong analyzed and interpreted the data. Huajiang Deng and Luotong Liu wrote the paper. All authors read and approved the final manuscript submitted for publication.

## Data Availability

The data that support the findings of this study are available from the corresponding author upon reasonable request.
